# Streptomyces sediminimaris sp. nov., a novel actinobacterium with anticancer potential isolated from mangrove sediments

**DOI:** 10.1099/ijsem.0.006811

**Published:** 2025-06-13

**Authors:** Nuttaporn Emthomya, Chusanajit Chuangrattanawan, Chananan Ngamcharungchit, Tepakorn Kongsaya, Pawina Kanchanasin, Wongsakorn Phongsopitanun, Chanwit Suriyachadkun, Sacha J. Pidot, Bungonsiri Intra

**Affiliations:** 1 Department of Biotechnology, Faculty of Science, Mahidol University, Bangkok 10400, Thailand; 2 Mahidol University-Osaka University Collaborative Research Center for Bioscience and Biotechnology (MU-OU: CRC), Faculty of Science, Mahidol University, Bangkok 10400, Thailand; 3 Department of Biochemistry and Microbiology, Faculty of Pharmaceutical Sciences, Chulalongkorn University, Bangkok 10330, Thailand; 4 Natural Products and Nanoparticles Research Unit (NP2), Chulalongkorn University, Bangkok 10330, Thailand; 5 Thailand Bioresource Research Center (TBRC), National Center for Genetic Engineering and Biotechnology (BIOTEC), National Science and Technology Development Agency (NSTDA), 113 Thailand Science Park, Pathum Thani 12120, Thailand; 6 Department of Microbiology and Immunology, Doherty Institute for Infection and Immunity, University of Melbourne, Melbourne, Victoria 3000, Australia

**Keywords:** anticancer activity, mangrove sediment, novel *Streptomyces*, phenotypic variants

## Abstract

Two marine actinomycete-like strains, MCC20^T^ and MCC57, were isolated from Chanthaburi (Thailand) mangrove sediment. Their taxonomic classifications were established through a polyphasic approach. Despite differences in colony morphotypes, genetic and chemotaxonomic analyses confirmed them as the same species within the genus *Streptomyces*. Both strains contained ll-diaminopimelic acid in their cell wall, with glucose, mannose, ribose and rhamnose identified as whole-cell sugars. Their phospholipid profile comprises phosphatidylethanolamine, diphosphatidylglycerol, phosphatidylglycerol, phosphatidylinositol and phosphatidylinositol mannoside. The predominant fatty acids were iso-C_15:0_, anteiso-C_15:0_, iso-C_16:0_ and anteiso-C_17:0_ with MK-9(H_8_) as the primary menaquinone, while MK-9(H_6_) and MK-9(H_4_) were unique to strain MCC57. Both strains exhibited anticancer activity against colorectal (HCT116) and lung (A549) cancer cells, with strain MCC20^T^ being more potent. Their 16S rRNA gene sequences showed 100% similarity, with 99.2% similarity to *Streptomyces fumigatiscleroticus* NBRC 12999^T^. Nevertheless, a phylogenomic tree placed them closer to *Streptomyces spinosirectus* CRSS-Y-16^T^, *Streptomyces plumbidurans* KC 17012^T^ and *Streptomyces spinosisporus* 7R016^T^. Nearly 100% average nucleotide identity (ANI) and digital DNA–DNA hybridization (dDDH) values highlighted the identity of strains MCC20^T^ and MCC57, while ANI (89.4%) and dDDH (35.5%) values were well below the respective 95 and 70% thresholds for related species. This supported their novelty. Based on their genotypes and phenotypes, strains MCC20^T^ (=NBRC 117131^T^=TBRC 19240^T^) and MCC57 (=NBRC 117132=TBRC 19241) are identified as phenotypic variants of a new species, *Streptomyces sediminimaris* sp. nov., with strain MCC20^T^ designated as the type strain (~9.2 Mb genome, 72.0 mol% G+C content).

## Introduction

*Streptomyces*, described by Waksman and Henrici in 1943, represent the largest group within the phylum *Actinomycetota*, with 741 validly published species listed in the List of Prokaryotic Names with Standing in Nomenclature (LPSN; https://lpsn.dsmz.de/genus/streptomyces) as of 24 February 2025 [1,2]. They are Gram-positive, sporulating filamentous bacteria that form highly branched substrate and aerial mycelia with diverse pigments, maturing into chains of three or more spores. The prevalence of ll-diaminopimelic acid in their cell wall peptidoglycan, along with the occasional presence of glucose, mannose and ribose in whole-cell hydrolysates, uniquely distinguishes *Streptomyces* from other genera in the *Streptomycestaceae* family [3]. Rarely found in other bacterial taxa, *Streptomyces* possess large linear chromosomes ranging from 6 to 15 Mb and have a high G+C content, averaging 72 mol%. Their genomes display genetic compartmentalization, highlighting stable core genes in the central region, flanked by fast-evolving arms that lead to genetic instability [4,5]. The dynamic chromosome arms drive rapid variability in *Streptomyces* morphology, physiology and secondary metabolism at both species and strain levels. This genetic flexibility directly influences the production of a wide range of bioactive compounds, including antibacterial, antifungal, antiparasitic and anticancer agents [6,7]. Although *Streptomyces* are widespread across terrestrial and aquatic ecosystems, with the highest diversity in soil [3,8], the frequent rediscovery of known compounds from soil species has led to a shift in research focus towards underexplored marine environments. Over the past three decades, the proportion of novel metabolites from marine *Streptomyces* has risen significantly, from 23.0 to 40.1% [9]. Among marine habitats, mangroves that serve as ecological bridges among land, sea and brackish water have been highly valuable for isolating new species that produce unique secondary metabolites [10,11]. These habitats are characterized by fluctuating pH, salinity and organic matter, creating rich biodiversity with diverse ecological niches. Such dynamic conditions favour *Streptomyces*, renowned for its adaptability, as the dominant bacterial genus yielding over 40% of new marine *Streptomyces* discoveries from 2015 to 2020 [12,13].

Southeast Asia has about one-third of the world’s mangroves, making it a global hotspot for this ecosystem [10]. Thailand, centrally situated in Southeast Asia, has high-density mangroves along the muddy tidal flats of the Upper Gulf of Thailand, including Chanthaburi province [14]. In 2019, our collection activities yielded over 300 culturable actinomycete strains isolated from various mangrove sediment sites in Chanthaburi. Among them, two strains, MCC20^T^ and MCC57, show unique colony appearances that are genetically related based on 16S rRNA gene sequences. So, we aim to taxonomically describe strains MCC20^T^ and MCC57 through a polyphasic approach while evaluating their potential to produce anticancer bioactive compounds.

## Isolation

Sediment samples were collected from four sites in a mangrove forest of Chanthaburi province (12° 31′ 51.6″ N, 102° 03′ 00.9″ E), Thailand, beginning on 16 May 2019. The air-dried samples were diluted with sterile normal saline and then spread onto actinomycete isolation media. Two isolates, MCC20^T^ and MCC57, were obtained on starch-casein-nitrate agar [15] supplemented with nalidixic acid (25 µg ml^−1^) and cycloheximide (50 µg ml^−1^) after incubation at 30 °C for 1–3 weeks. Pure cultures of these isolates were routinely preserved in International *Streptomyces* Project (ISP) 2 medium [16] with 20% (v/v) glycerol at −80 °C for long-term storage.

## 16S rRNA gene sequencing and phylogeny

To prepare cell biomass used for a genotypic study, strains MCC20^T^ and MCC57 were cultivated in tryptic soy broth (TSB; Difco, USA) at 30 °C with shaking at 200 r.p.m. After 3 days of culture, cells were harvested, washed and freeze-dried. Genomic DNA (gDNA) was extracted using the method of Saito and Miura [17], with slight adaptations. In the modified lysis process, freeze-dried cells were physically disrupted by grinding in a liquid nitrogen-cooled mortar before overnight enzymatic treatment with mutanolysin (Merck, Germany), achromopeptidase (Merck, Germany) and lysozyme (Thermo Fisher Scientific^™^, USA) [18,20]. The gDNA products were PCR-amplified in the region of the 16S rRNA gene using 27F and 1492R universal primers [21,22]. The PCR products were Sanger sequenced (Macrogen, Korea), and the nearly full-length 16S rRNA gene sequences were compared for pairwise similarity against the taxa list in the EzBioCloud database, available at https://www.ezbiocloud.net/ [23]. Their close phylogenetic neighbours were selected for studying evolutionary relationships by phylogenetic tree construction. The 16S rRNA gene sequences of strains MCC20^T^ and MCC57, together with their related species, were aligned using the muscle method [24]. Evolutionary trees were built based on the neighbour-joining (NJ), minimum-evolution (ME) and maximum-likelihood (ML) algorithms, as implemented in MEGA X [25]. The evolutionary distance in terms of nucleotide substitution within the 16S rRNA gene sequence was analysed using the Kimura two-parameter model [26]. Bootstrapping with 1,000 replicates was employed to assess the confidence level of the tree’s robustness [27].

**Fig. 1.**
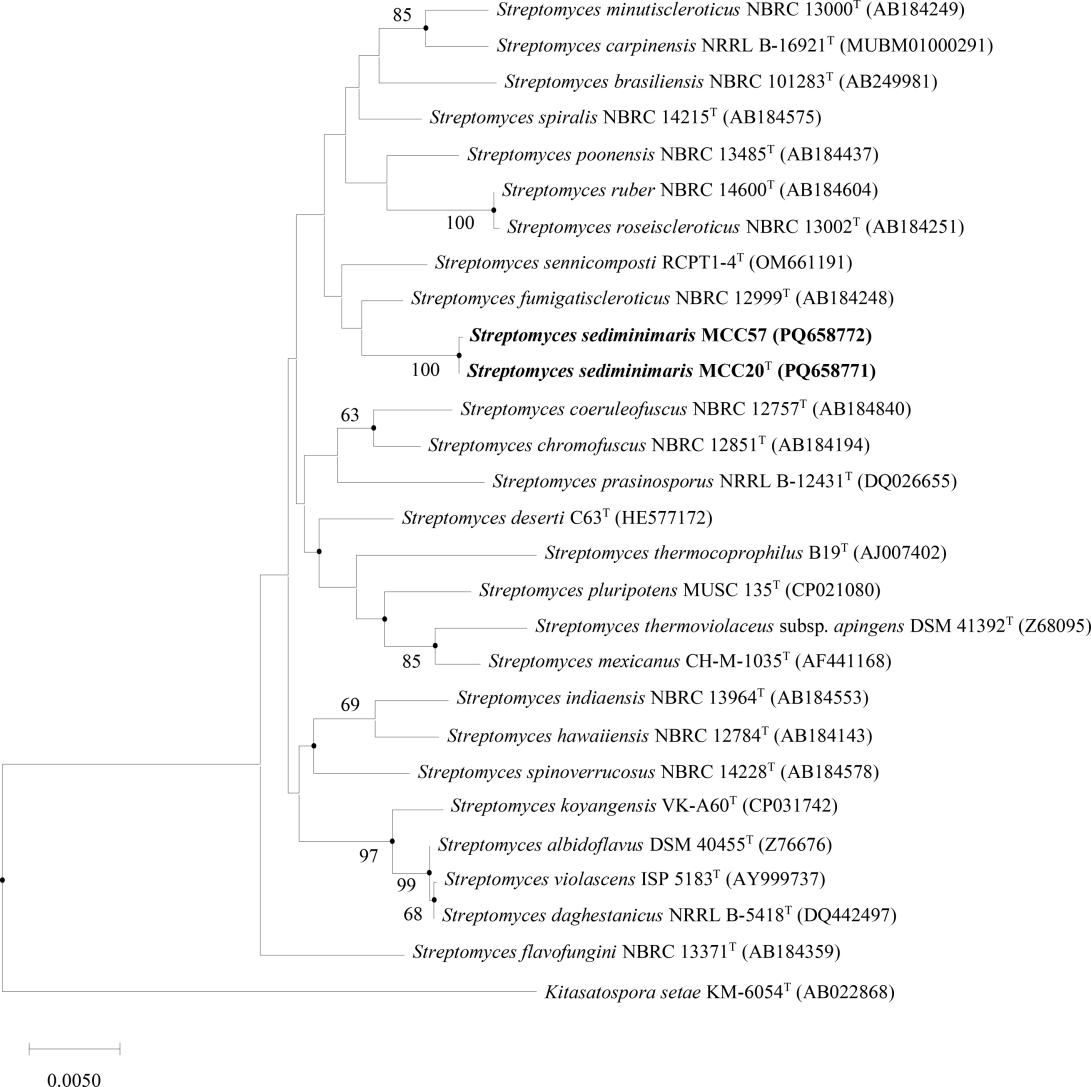
16S rRNA gene-based phylogeny built by the NJ algorithm. Genetic relationships among strains MCC20^T^, MCC57 and their closely related type strains are shown, with *Kitasatospora setae* KM-6054^T^ (AB022868) serving as the outgroup. Bootstrap values >50% from 1,000 replications are displayed above the branches. The branch lengths are scaled to represent 0.005 substitutions per nucleotide position. Dots indicate branches that are also supported by the ME and ML trees (see Figs S1and S2).

Based on almost complete 16S rRNA gene sequence, MCC20^T^ (1,472 bp) and MCC57 (1,418 bp) were submitted to the GenBank^®^ database (PQ658771 and PQ658772, respectively), with all hit taxa belonging to the genus *Streptomyces*. The blastn result showed 99.58% similarity between these two strains. Likewise, strains MCC20^T^ and MCC57 have an identical closest related species, *Streptomyces fumigatiscleroticus* NBRC 12999^T^ (99.2%) followed by *Streptomyces sennicomposti* RCPT1-4^T^ (98.8%) and *Streptomyces spiralis* NBRC 14215^T^ (98.8%). There is evidence suggesting a species-level similarity between strains MCC20^T^ and MCC57, as indicated by the 98.65% threshold typically employed to differentiate species [28]. However, this threshold requires the calculation of overall genomic relatedness indices (OGRIs) for confirmation. Commonly used values for species delineation and phylogenetic position of a new species include digital DNA–DNA hybridization (dDDH) and average nucleotide identity (ANI) [29,30]. Consistent with 16S rRNA gene sequence similarity, strains MCC20^T^ and MCC57 were positioned on the same node with 100% bootstrap support, indicating strong confidence in their genetic relationship (Figs 1, S1 and S2, available in the online Supplementary Material). This suggests they are the closest relatives, likely belonging to the same species. Both strains were closely clustered with *S. fumigatiscleroticus* NBRC 12999^T^ in a distinct group, while *S. sennicomposti* RCPT1-4^T^ formed a separate branch with a weak bootstrap value (<50%). These strains show a monophyletic group sharing a common ancestor.

## Genome features and phylogenomy

Extracted gDNA of strains MCC20^T^ and MCC57 was subjected to whole genome sequencing with hybrid long-read and short-read assembly. Short-read sequencing was performed using the DNBseq^™^ platform (BGI, Hong Kong) and the Illumina NextSeq 550 system (Celemics, Inc., Korea). Simultaneously, extracted gDNA was prepared for sequencing on the Oxford Nanopore MinION platform according to the protocol of the Ligation Sequencing Kit (Oxford Nanopore). DNA samples were loaded onto a single MinION flow cell and data were collected over 72 h. Unicycler (version 0.4.8) was used to perform hybrid assemblies using long and short reads. The whole genomes of strains MCC20^T^ and MCC57, along with 16S rRNA gene fragments extracted from the genomes, were deposited into the GenBank^®^ database. Genome assembly quality, including N50, L50, contig count, genome size and G+C (mol%) content, was evaluated using the quality assessment tool QUAST (version 5.2.0) [31]. This was followed by genomic feature prediction with Prokka (version 1.14.6) [32]. Additionally, biosynthetic gene clusters (BGCs) were identified using antiSMASH (version 7.0) [33]. To complete genome-based prokaryote taxonomy, *in silico* (sub)species delineation was conducted based on dDDH and ANI values. They were simultaneously calculated with genome-based phylogenetic reconstruction to determine intra- or inter-species relationships [34]. The dDDH index and phylogenomic tree were automatically analysed using the genome-to-genome distance calculator (GGDC version 3.0) on the type (strain) genome server (TYGS) web server, accessible at https://tygs.dsmz.de/ [35]. A multilocus sequence analysis (MLSA) phylogenetic tree was also built using the autoMLST server (https://automlst.ziemertlab.com/) [36]. Type strains closely related to MCC20^T^ and MCC57 were selected for pairwise genome comparisons, shown in ANI values. The ANI-blast+ (ANIb) and ANI-MUMmer (ANIm) values were achieved using JSpeciesWS, available at https://jspecies.ribohost.com/jspeciesws/ [37]. The dDDH value (computed by GGDC formula 2, which is preferred for dealing with incomplete genomes), ANI values and phylogenomy were all essential for identifying potential new species [34,38].

**Fig. 2.**
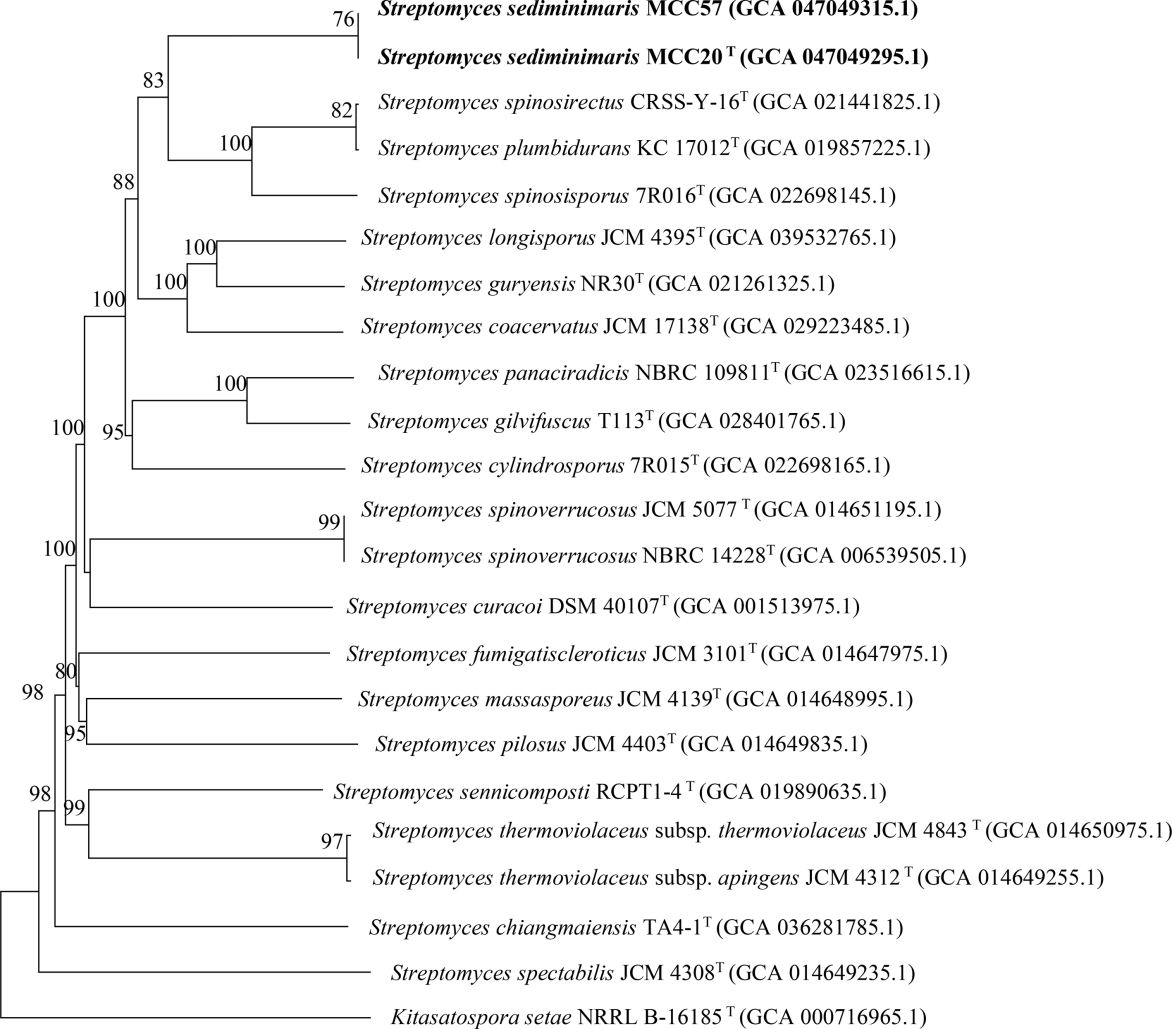
Genome-based phylogeny of strains MCC20^T^ and MCC57 inferred with the FastME 2.1.6.1 algorithm [65] based on genome blast distance phylogeny (GBDP) distances automatically calculated by TYGS. *Kitasatospora setae* NRRL B-16185^T^ (GCA 000716965.1) is applied as the outgroup. GBDP pseudo-bootstrap values >60% from 100 replications are shown above the branches, with the branch lengths scaled according to the GBDP distance formula *d_5_*.

The entire genomes of MCC20^T^ (JBJWOT000000000) and MCC57 (JBJWOU000000000) are publicly available at NCBI. According to hybrid genome assembly, the MCC20^T^ genome has a 9,180,064 bp length (number of contigs, 6; contig N50, 8,327,590 bp; contig L50, 1) with a DNA G+C content of 72.0 mol%. The genome size of MCC57 is 9,017,300 bp (number of contigs, 24; contig N50, 1,471,545 bp; contig L50, 3) with 72.1 mol% G+C. They harbour a large genome that is ~72 mol% G+C rich, which is characteristic of *Streptomyces* populations [4]. In genome-scale phylogeny, strains MCC20^T^ and MCC57 form a branch within the same independent group supported by a 76% bootstrap value, suggesting these two isolates have the nearest evolutionary relationship (Fig. 2). Inconsistent with 16S rRNA gene-based phylogeny, they cluster closely with a monophyletic group of *Streptomyces spinosirectus* CRSS-Y-16^T^, *Streptomyces plumbidurans* KC 17012^T^ and *Streptomyces spinosisporus* 7R016^T^ with a bootstrap value of 83%. All species originating from this single lineage share a recent common ancestor. The *S. fumigatiscleroticus* JCM 3101^T^, a 16S rRNA gene-based related taxon, lies in a distant clade showing genetic divergence from the MCC20^T^ and MCC57 lineages. Nevertheless, genome assembly of *S. fumigatiscleroticus* JCM 3101^T^ was suppressed from the RefSeq database because of contamination found by the CheckM automated tool [39]; see https://www.ncbi.nlm.nih.gov/datasets/genome/GCF_014647975.1/. A contaminated genome can lead to false taxonomic classification [40]. Based on the MLSA phylogenetic tree, strains MCC20^T^ and MCC57 formed a distinct group with 100% bootstrap support, confirming their nearest neighbours branched independently from another monophyletic *Streptomyces* group (Fig. S3). Closely related species in the monophyletic group differed from those of strains MCC20^T^ and MCC57 as determined by 16S rRNA gene and genomic sequences. Considering 16S rRNA gene and genome-based phylogenetic trees, *S. fumigatiscleroticus* JCM 3101^T^, *S. plumbidurans* KC 17012^T^, *S. spinosirectus* CRSS-Y-16^T^ and *S. spinosisporus* 7R016^T^ were chosen as reference species for pairwise comparison with strains MCC20^T^ and MCC57 (Table 1). All OGRIs between the whole genomes of MCC20^T^ and MCC57 are nearly 100%. Since 70% dDDH and 95–96% ANI are cut-off thresholds for bacterial species delineation [41,42], MCC20^T^ and MCC57 are the same species. However, they and their three closest phylogenomic neighbours share the highest dDDH and ANI indices, which are significantly lower than the minimum threshold. Consequently, the two intra-species, strains MCC20^T^ and MCC57, are considered a new species of the genus *Streptomyces*.

**Table 1.** Pairwise genome sequence comparisons among strains MCC20^T^, MCC57 and their closely related strains

Species 1 (GenBank ID)	Species 2 (GenBank ID)	G+C difference	dDDH	ANIb	ANIm
*Streptomyces* sp. MCC20^T^ (GCA 047049295.1)	*Streptomyces* sp. MCC57 (GCA 047049315.1)	0.1	99.9	99.9	100.0
*Streptomyces* sp. MCC20^T^ (GCA 047049295.1)	*S. fumigatiscleroticus* JCM 3101^T^ (GCA 014647975.1)	0.5	27.2	81.7	86.6
*Streptomyces* sp. MCC20^T^ (GCA 047049295.1)	*S. spinosirectus* CRSS-Y-16^T^ (GCA 021441825.1)	1.3	35.5	87.3	89.4
*Streptomyces* sp. MCC20^T^ (GCA 047049295.1)	*S. plumbidurans* KC 17012^T^ (GCA 019857225.1)	1.2	35.5	87.3	89.4
*Streptomyces* sp. MCC20^T^ (GCA 047049295.1)	*S. spinosisporus* 7R016^T^ (GCA 022698145.1)	1.3	35.6	87.2	89.4
*Streptomyces* sp. MCC57 (GCA 047049315.1)	*S. fumigatiscleroticus* JCM 3101^T^ (GCA 014647975.1)	0.5	27.2	81.7	86.6
*Streptomyces* sp. MCC57 (GCA 047049315.1)	*S. spinosirectus* CRSS-Y-16^T^ (GCA 021441825.1)	1.3	35.5	87.4	89.4
*Streptomyces* sp. MCC57 (GCA 047049315.1)	*S. plumbidurans* KC 17012^T^ (GCA 019857225.1)	1.2	35.5	87.4	89.4
*Streptomyces* sp. MCC57 (GCA 047049315.1)	*S. spinosisporus* 7R016^T^ (GCA 022698145.1)	1.3	35.5	87.3	89.4

According to antiSMASH results, the genome assemblies of MCC20^T^ and MCC57 contain several types of secondary metabolite biosynthetic gene clusters (smBGCs). The most abundant predicted smBGCs encode polyketide synthases (type I, type II and type III PKSs), followed by terpenes, ribosomally synthesized and post-translationally modified peptides (RiPPs), NI-siderophore, hybrid non-ribosomal peptide synthetase–PKS (NRPS/PKS), butyrolactone, NRPS, ectoine and melanin (Fig. S4). We found additional type I PKS and RiPP BGCs in the MCC57 genome, distinguishing it from MCC20^T^. Insights from the *Streptomyces* pangenome reveal that many smBGCs, particularly PKSs and NRPS, are strain-specific, supporting metabolomic diversity that varies in identical species [6,7, 43].

## Morphology

To examine the cultural characteristics of strains MCC20^T^ and MCC57, 3-day-old cultures were washed twice and streaked onto ISP media, following the guidelines established by Shirling and Gottlieb [16]. The appearance of fully mature colonies grown on ISP 2, ISP 3, ISP 4, ISP 5, ISP 6 and ISP 7 (Difco, USA; HiMedia, India) agar was recorded after incubation at 30 °C for 14 days. Key characteristics include observable growth levels, colours of colonies, spore production and soluble pigments which were designated based on the Inter-Society Color Council National Bureau of Standards colour chart [44]. Additionally, spore morphologies were observed under scanning electron microscopy (JSM-6610LV; JEOL) operated by the Scientific and Technological Research Equipment Centre, Chulalongkorn University (Bangkok, Thailand). These characteristics of strains MCC20^T^ and MCC57 were compared with chosen reference strains, including *S. fumigatiscleroticus* TBRC 14820^T^ and *S. spinosisporus* 7R016^T^.

**Fig. 3.**
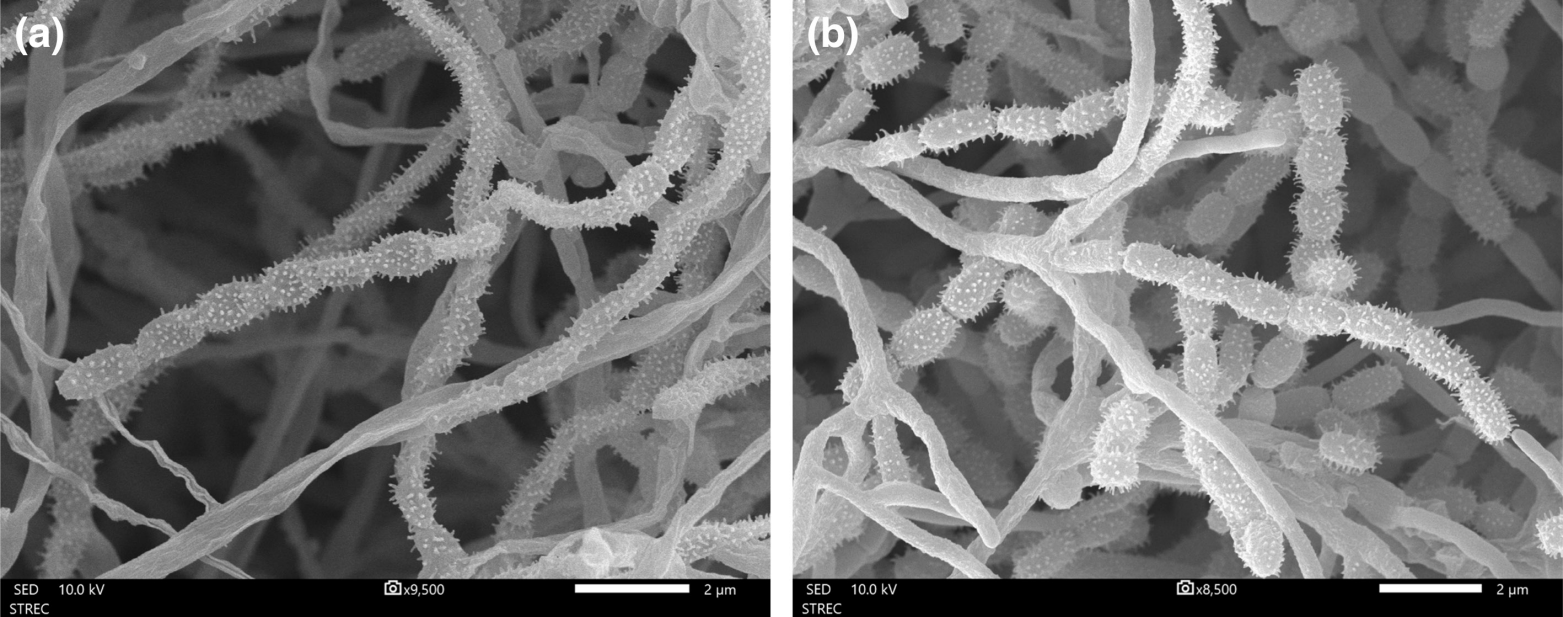
Scanning electron micrographs visualizing the polysporous chains of strains (a) MCC20^T^ and (b) MCC57 grown on ISP 3 agar at 30 °C for 14 days of incubation. Scale bar, 2 µm.

Given their identical genetic data, phenotypic traits and differences in colony morphology were crucial for distinguishing strains MCC20^T^ and MCC57. They grew well on ISP 2 to ISP 7 media, producing yellow soluble pigments, except on ISP 6 for strain MCC20^T^ and ISP 6–7 for strain MCC57. Their colony colours varied distinctly across the media, highlighting observable differences between the two strains. Aerial mycelia of strain MCC20^T^ exhibited a range of colours from light orange-yellow to strong reddish-orange. In contrast, strain MCC57 showed various reddish shades observed on ISP 2 to ISP 5 media, with pale orange-yellow colonies on ISP 6 and ISP 7. The colony appearance of strains MCC20^T^ and MCC57 differed from their reference strains in both colour shades and the ability to produce soluble pigments (Table S1). Strain MCC20^T^ produced spores on ISP 2–ISP 4, while strain MCC57 formed white spores exclusively on ISP 3, with both strains exhibiting poor spore production. Consistent with other *Streptomyces* species, the mature spores of these strains exhibited similar morphologies, forming straight, branched polysporous chains with spiny cylindrical surfaces (Fig. 3). The early-stage spores, in contrast, displayed smooth surfaces (Fig. S5). Most spores measured ~0.5–0.8 µm in width and 0.9–1.4 µm in length. According to these results, macroscopic morphology like colony appearance could be used to distinguish between strains MCC20^T^ and MCC57. Their spore morphologies were quite similar, with *S. spinosisporus* 7R016^T^ presenting long chains of spiny-type spores [45]. In contrast, they were different from *S. fumigatiscleroticus* NBRC 12999^T^, carrying spiral spore chains with smooth spore surfaces (available at https://bacdive.dsmz.de/strain/15209).

## Physiology

The growth capabilities of the strains were assessed on ISP 2 basal agar under various temperatures (4, 20, 25, 30, 37, 40 and 45 °C), pH levels from 3.0 to 11.0 (in 1.0-unit increments) and NaCl concentrations from 1.0 to 10.0% (w/v, in 1.0% intervals). Carbon source utilization was assessed on an ISP 9 medium supplemented with various carbon sources at a final concentration of 1.0% (w/v) [16]. Physiological traits, including starch hydrolysis, H_2_S production, tyrosinase activity and nitrate reduction, were evaluated using ISP 4, ISP 6, ISP 7 and ISP 8 media, respectively [16,46,48]. Casein hydrolysis and gelatin liquefaction were analysed using 10% skim milk and peptone-glucose-gelatin media, respectively [49]. All tests were conducted at 30 °C, and results were recorded after 14 days of incubation. Furthermore, enzyme activity and fermentation capability were determined using 3-day-old cultures grown in TSB medium and tested with a commercial API ZYM test kit (bioMérieux, France) following the manufacturer’s instructions. The physiological traits of *S. fumigatiscleroticus* TBRC 14820^T^ and *S. spinosisporus* 7R016^T^, closely related strains, were also described to assess the novelty of strains MCC20^T^ and MCC57.

In replicate experiments, the optimal growth temperature for strains MCC20^T^ and MCC57 was 30 °C, with slower growth at 40 °C. Strain MCC20^T^ demonstrated pH adaptability, thriving at pH 4.0–11.0, while strain MCC57 was slightly more fastidious, growing between pH 5.0 and 11.0. Both strains exhibited optimal growth at pH 7.0. Strain MCC20^T^ tolerated up to 9% (w/v) NaCl, whereas strain MCC57 exceeded this value, growing even at the maximum tested concentration, 10% (w/v) NaCl. The strains utilized in the current study all used monosaccharides (l-arabinose, d-glucose, d-mannitol, d-mannose, rhamnose and d-xylose) and disaccharides (maltose and sucrose) in ISP 9 agar as sole carbon sources. They also presented positive results for starch hydrolysis and nitrate reduction, as well as milk peptonization and coagulation. However, they tested negative for H_2_S production, tyrosinase activity and gelatin liquefaction. Of the 19 reactions tested in the API ZYM, strains MCC20^T^ and MCC57 could produce most of the enzymes of interest, except for lipase and *β*-glucuronidase. The physiological characteristics of strains MCC20^T^ and MCC57, compared with their reference species, are detailed in Table 2. Despite their genetic similarities, strains MCC20^T^ and MCC57 exhibited slight phenotypic variations, particularly in pH and NaCl tolerance. Their optimal growth conditions were similar to closely related taxa, but they differed from *S. fumigatiscleroticus* TBRC 14820^T^ in tyrosinase activity, nitrate reduction and enzymatic profiles. Moreover, strains MCC20^T^ and MCC57 showed negative gelatin liquefaction and positive sucrose utilization, unlike *S. spinosisporus* 7R016^T^ phenotypes. These distinctive physiological traits contribute to their novelty among recognized *Streptomyces* species.

**Table 2.** Differential physiological characteristics among strains MCC20^T^, MCC57 and their closely related strains Strain: 1, MCC20^T^; 2, MCC57; 3, *S. fumigatiscleroticus* TBRC 14820^T^; 4, *S. spinosisporus* 7R016^T^. Optimal growth conditions are indicated in parentheses. +, positive; w, weakly positive; −, negative; nd, not determined.

Characteristic	1	2	3	4†
Growth temperature (°C)	20–40 (30)	20–40 (30)	20–45 (30)	25–40 (30)
Growth pH	4.0–11.0 (7.0)	5.0–11.0 (7.0)	4.0–11.0 (7.0)	6.0–10.0 (7.0)
NaCl tolerance (%, w/v)	0–9 (0–4)	0–10 (0–5)	0–6 (0)*	0–8 (nd)
Starch hydrolysis	+	+	+	+
H_2_S production	–	–	–	nd
Tyrosinase activity	–	–	+	nd
Nitrate reduction	+	+	–	+
Milk peptonization/coagulation	+/+	+/+	+/+	−/+
Gelatin liquefaction	–	–	–	+
**Carbon source utilization**				
l-Arabinose	+	w	+	w
d-Glucose	+	+	+	nd
d-Mannitol	+	+	+	w
d-Mannose	w	w	+	+
Rhamnose	+	w	+	+
d-Xylose	w	+	+	+
Maltose	w	+	+	nd
Sucrose	+	+	w	–
**API ZYM (enzyme activity)**				
Alkaline phosphatase	w	w	+	nd
Esterase (C4)	w	w	–	nd
Esterase lipase (C8)	w	w	–	nd
Lipase (C14)	–	–	–	nd
Leucine arylamidase	+	+	+	nd
Valine arylamidase	+	+	+	nd
Cystine arylamidase	+	+	+	nd
Trypsin	+	+	+	nd
*α*-Chymotrypsin	+	+	w	nd
Acid phosphatase	+	+	+	nd
Naphthol-AS-BI-phosphohydrolase	+	+	+	nd
*α*-Galactosidase	+	+	+	nd
*β*-Galactosidase	+	+	+	nd
*β*-Glucuronidase	–	–	–	nd
*α*-Glucosidase	+	+	+	nd
*β*-Glucosidase	+	+	+	nd
*N*-Acetyl-*β*-glucosaminidase	+	+	+	nd
*α*-Mannosidase	+	+	w	nd
*α*-Fucosidase	+	+	–	nd

*Data are obtained from the BacDrive entry for *S. fumigatiscleroticus* 145 (=NBRC 12999T).

†Features are provided in the *IJSEM* article by Kanchanasin *et al*. [45].

## Chemotaxonomy

Freeze-dried MCC20^T^ and MCC57 cells were prepared for chemotypic characterization. TLC was employed to detect the profile of diaminopimelic acid (DAP) isomers, whole-cell sugars and phospholipids. DAP isomers and whole-cell sugars were detected following the procedure of Staneck and Roberts [50]. Their phospholipid patterns were investigated using two-dimensional TLC (2D-TLC) sprayed with reagents according to Minnikin *et al*. [51]. Cell hydrolysates were extracted using the method of Collins *et al*. [52] to study menaquinone composition. Isoprenoid quinone extracts were then analysed using HPLC eluted in an isocratic mode with methanol and 2-propanol (2:1, v/v), at a 1 ml min^−1^ flow rate, with UV absorbance at 270 nm. Organic solvent extraction and GC analysis were conducted using the Sherlock Microbial Identification System (MIDI) [53], Sherlock Version 6.4 and RTSBA6 database for cellular fatty acid analysis. The fatty acid profiles of *S. fumigatiscleroticus* TBRC 14820^T^ and *S. spinosisporus* 7R016^T^ were also considered.

Strains MCC20^T^ and MCC57 contained ll-diaminopimelic acid, predominantly found in their cell walls [54]. Glucose, mannose, ribose and rhamnose were detected in whole-cell hydrolysates. Phosphatidylethanolamine (PE), diphosphatidylglycerol (DPG), phosphatidylglycerol (PG), phosphatidylinositol (PI), phosphatidylinositol mannoside (PIM) and one unidentified phospholipid were observed in strains MCC20^T^ and MCC57 (Figs S6 and S7) based on phospholipid spots on 2D-TLC. The type II phospholipid profile was suggested for these strains due to the presence of PE, DPG, PG, PI and PIM with no phosphatidylcholine, consistent with the common polar lipid markers of *Streptomyces* [55]. The main menaquinone of strain MCC20^T^ was MK-9(H_8_) with 100% abundance, while strain MCC57 contained MK-9(H_8_), MK-9(H_6_) and MK-9(H_4_) with 56.93, 35.46 and 7.61% abundance, respectively. MK-9(H_6_) and MK-9(H_4_) were unique to strain MCC57. The predominance of MK-9(H_4_, H_6_ and H_8_) is a hallmark feature of *Streptomyces* [56]. In fatty acid profiling, the G+C peak abundance found that at least 10 mol% was composed of major fatty acids. Strain MCC20^T^ was comprised of iso-C_16:0_ (22.4%), iso-C_15:0_ (16.8%) and anteiso-C_15:0_ (16.5%), whereas strain MCC57 showed anteiso-C_15:0_ (20.2%), iso-C_16:0_ (18.5%) and anteiso-C_17:0_ (16.0%). They held iso-C_(15:0, 16:0, 17:0)_ and anteiso-C_(15:0, 17:0)_ typically present in the genus *Streptomyces* [56]. A comparison of the major and minor fatty acids between these two strains and their closely related species revealed distinct compositions (Table S2).

## *In vitro* evaluation of anticancer activity

Three-day-old cultures of strains MCC20^T^ and MCC57 were inoculated into 100 ml of a 301 production medium (0.1% glucose, 2.4% soluble starch, 0.3% meat extract, 0.3% peptone, 0.5% yeast extract, 0.4% CaCO_3_, pH 7.0) with a 5% inoculum. These seeded media were incubated for 7 days at 30 °C with shaking at 200 r.p.m. Crude extracts were obtained after liquid–liquid extraction of supernatant and cell pellets with ethyl acetate and ethanol, respectively. All MCC20^T^, MCC57 and 301 extracts were dissolved in DMSO (Merck, Germany) for high-throughput anticancer activity screening using the 3-[4,5-dimethylthiazol-2-yl]-2,5 diphenyl tetrazolium bromide (MTT) assay [57]. A final concentration of 10 µg ml^−1^ crude extracts was tested for activity against colorectal (HCT116; ATCC, USA) and lung (A549; ATCC, USA) cancer cell lines. Doxorubicin (10 µg ml^−1^; Sigma-Aldrich, USA) and cisplatin (10 µg ml^−1^; Sigma-Aldrich, USA) were used as positive controls for HCT-116 and A549 cells, respectively, while DMSO (0.5%; Merck, Germany) served as the negative control. After incubating cancer cells under CO_2_ for 72 h with test agents, cell viability was measured and the percentage of cell inhibition was calculated.

A crude extract of strain MCC20^T^ presented potent anticancer activity, inhibiting over 99% of HCT116 colorectal and A549 lung cancer cells, whereas strain MCC57 showed weak activity (<80% inhibition) against the same cancer cell lines (Table 3). Remarkably, the efficacy of the strain MCC20^T^-derived extract surpassed that of the chemotherapeutic drugs doxorubicin [58] and cisplatin [59]. MTT assay results indicated that the 301 medium, which inhibited <30% of cancer cells, did not significantly contribute to the observed anticancer effects. Based on the bioactivity results, the smBGCs responsible for synthesizing anticancer metabolites in strains MCC20^T^ and MCC57 were extensively analysed. Genomic analysis of both strains revealed four smBGCs (>70% similarity) associated with anticancer activity, including *ε*-poly-l-lysine [60], desferrioxamine [61], actinomycin D [62] and undecylprodigiosin [63]. These clusters belong to NAPAA, NI-siderophore, NRPS and NRPS/PKS biosynthetic types, respectively. Despite this similarity, only strain MCC20^T^ displayed strong anticancer activity, likely due to differences in cluster-situated regulatory genes that control smBGC expression and are often poorly conserved [7,64]. This suggests that some anticancer-related smBGCs in strain MCC57 were weakly expressed under 301 medium conditions. Future metabolomic and transcriptomic analyses will explore this hypothesis.

**Table 3.** Anticancer activities of crude extracts of strains MCC20^T^ and MCC57

Crude extract	% Cancer cell inhibition	
	HCT116 colorectal cancer cells	A549 lung cancer cells
301 medium	25.6	25.8
MCC20^T^	99.3	99.2
MCC57	28.8	71.6
**Positive control**		
Doxorubicin	92.1	–
Cisplatin	–	94.3

–, not performed.

In line with polyphasic taxonomic results, genetic features (OGRIs, 16S rRNA gene- and genome-based phylogenies) of strains MCC20^T^ and MCC57 show that they belong to the same species, despite differences in their phenotypic and chemotypic traits. They vary in cultural morphologies on ISP media, growth pH, NaCl tolerance, phospholipid patterns, menaquinone composition, as well as fatty acid profiles. In terms of bioactivity, the crude extract of strain MCC20^T^ showed significantly stronger activity against colorectal (HCT116) and lung (A549) cancer cell lines compared to strain MCC57, though both strains harbour similar anticancer-related smBGCs in their genomes. Comparing the closest phylogenetic neighbours, greatly lower than 70% dDDH and 95–96% ANI indices, together with dissimilar phenotypes of strains MCC20^T^ and MCC57, supported their potential as new species. All chemotaxonomic characteristics suggested that these two strains are members of the genus *Streptomyces*. Collectively, they could be distinguished from the recognized *Streptomyces* species based on genetic, phenotypic and chemotaxonomic characteristics. Therefore, we propose strains MCC20^T^ and MCC57 as phenotypic variants of a novel *Streptomyces* species, designated as *Streptomyces sediminimaris* sp. nov.

## Description of *Streptomyces sediminimaris* sp. nov.

*Streptomyces sediminimaris* (se.di.mi.ni.ma’ris. L. neut. n. *sedimen*, sediment; L. neut. n. *mare*, the sea; N.L. gen. n. *sediminimaris*, of marine sediment).

An aerobic filamentous actinobacterium develops substrate and aerial mycelia with straight, branched, polysporous chains of spiny spores measuring 0.5–0.8 µm×0.9–1.4 µm. Its phenotypic traits are strain-specific. The type strain grows well on ISP (2–7) media, forming colonies in shades from light orange-yellow to reddish-orange, and typically secretes yellow soluble pigments, except on ISP 6. Growth occurs at 20–40 °C, pH 4.0–11.0 and up to 9% (w/v) NaCl. It utilizes l-arabinose, d-glucose, d-mannitol, d-mannose, rhamnose, d-xylose, maltose and sucrose as sole carbon sources on ISP 9 agar. Starch hydrolysis, nitrate reduction, milk peptonization and milk coagulation are positive in this species, while H_2_S production, tyrosinase activity and gelatin liquefaction are negative. Most enzymes tested on API ZYM show positive reactions, except for lipase and *β*-glucuronidase. The cell wall peptidoglycan contains ll-diaminopimelic acid with glucose, mannose, ribose and rhamnose in whole-cell hydrolysates. The predominant fatty acids are iso-C_15:0_, anteiso-C_15:0_ and iso-C_16:0_ with MK-9(H_8_) as the major menaquinone. Its phospholipid profile comprises PE, DPG, PG, PI and PIM.

Two phenotypic variants are MCC20^T^ (=NBRC 117131^T^=TBRC 19240^T^) and MCC57 (=NBRC 117132=TBRC 19241) isolated from mangrove sediment in Chanthaburi, Thailand. With its higher genome quality, the type strain is MCC20^T^. The genome assembly has a 9,180,064 bp size with a DNA G+C content of 72.0 mol%. The GenBank accession number for this genome is JBJWOT000000000 with PQ658771 for the 16S rRNA gene.

## Supplementary material

10.1099/ijsem.0.006811 Uncited Supplementary Material 1.

## Abbreviations

ANI: average nucleotide identity
BGCs: biosynthetic gene clusters
DAP: diaminopimelic acid
dDDH: digital DNA–DNA hybridization
DPG: diphosphatidylglycerol
GBDP: genome blast distance phylogeny
gDNA: genomic DNA
ISP: International *Streptomyces* Project
LPSN: List of Prokaryotic Names with Standing in Nomenclature
ME: minimum-evolution
ML: maximum-likelihood
MLSA: multilocus sequence analysis
MTT: 3-[4,5-dimethylthiazol-2-yl]-2,5 diphenyl tetrazolium bromide
NJ: neighbour-joining
NRPS: non-ribosomal peptide synthetase
OGRIs: overall genomic relatedness indices
PE: phosphatidylethanolamine
PG: phosphatidylglycerol
PI: phosphatidylinositol
PIM: phosphatidylinositol mannoside
PKS: polyketide synthase
RiPPs: ribosomally synthesized and post-translationally modified peptides
smBGC: secondary metabolite biosynthetic gene cluster
TSB: tryptic soy broth
TYGS: type (strain) genome server
