# Supplementary material for: Streptomyces sediminimaris sp. nov., a novel actinobacterium with anticancer potential isolated from mangrove sediments
Source: Int J Syst Evol Microbiol. 2025 Jun 13;75(6):006811. doi: 10.1099/ijsem.0.006811 (PMC12166134; doi:10.1099/ijsem.0.006811)
Supplement: Uncited Supplementary Material 1. [file ijsem-75-06811-s001.pdf]

Streptomyces sediminimaris **sp. nov., a novel actinobacterium with anticancer potential isolated from mangrove sediments**

Nuttaporn Emthomya[1,2], Chusanajit Chuangrattanawan[1,2], Pawina Kanchanasin[3], Wongsakorn Phongsopitanun[3,4], Chanwit Suriyachadkun[5], Sacha J. Pidot[6], Bungonsiri Intra[1,2,*]

[1] Department of Biotechnology, Faculty of Science, Mahidol University, Bangkok 10400, Thailand

[2] Mahidol University-Osaka University Collaborative Research Center for Bioscience and Biotechnology (MU-OU: CRC), Faculty of Science, Mahidol University, Bangkok 10400, Thailand

[3] Department of Biochemistry and Microbiology, Faculty of Pharmaceutical Sciences, Chulalongkorn University, Bangkok 10330, Thailand

[4] Natural Products and Nanoparticles Research Unit (NP2), Chulalongkorn University, Bangkok 10330, Thailand

[5] Thailand Bioresource Research Center (TBRC), National Center for Genetic Engineering and Biotechnology (BIOTEC), National Science and Technology Development Agency (NSTDA), 113 Thailand Science Park, Pathum Thani 12120, Thailand

[6] Department of Microbiology and Immunology, Doherty Institute for Infection and Immunity, University of Melbourne, Melbourne, Victoria 3000, Australia

Corresponding author: Bungonsiri Intra, bungonsiri.int@mahidol.edu

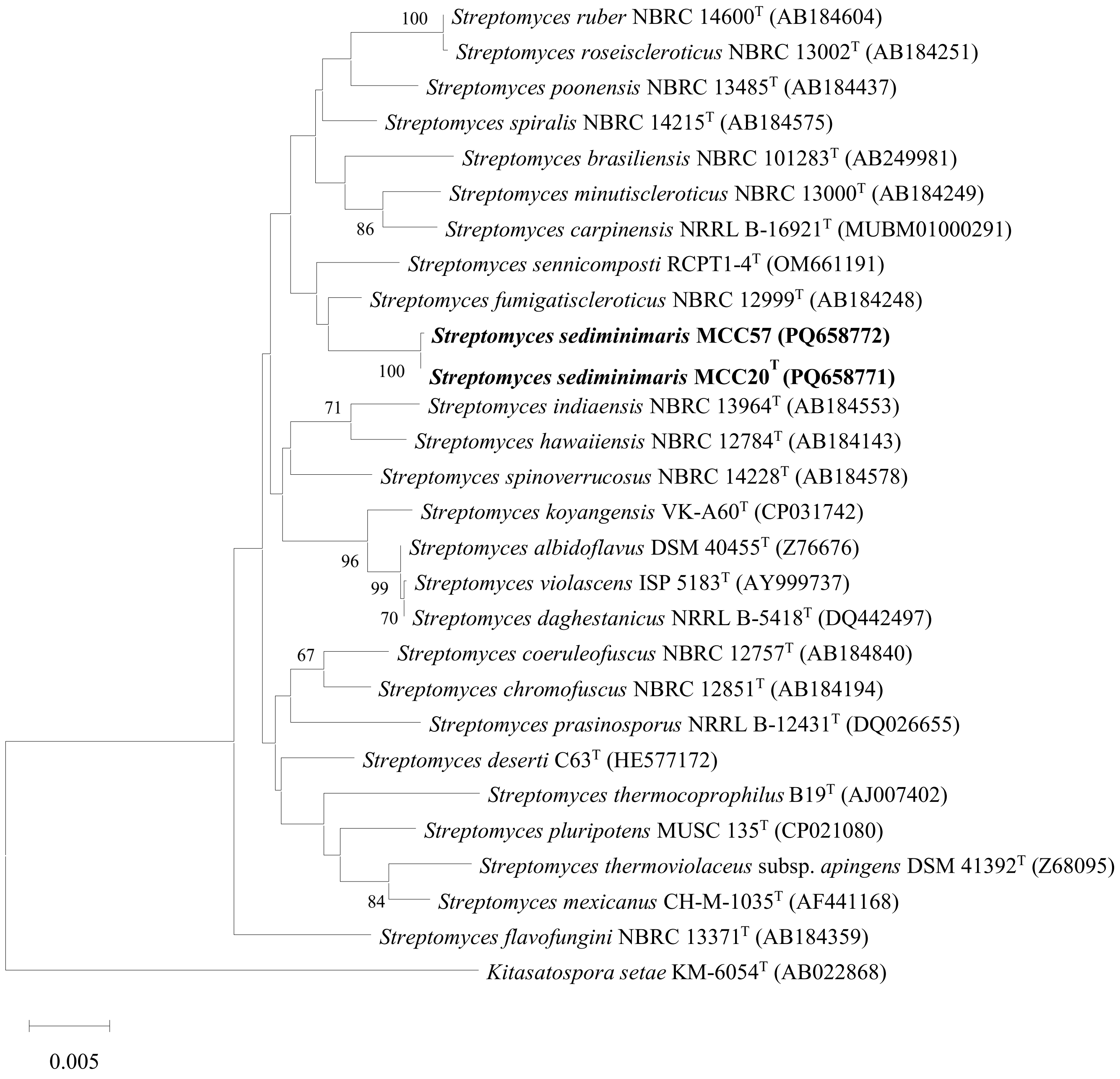

Fig. S1 16S rRNA gene-based phylogeny built by the Minimum-Evolution (ME) algorithm. Genetic relationships among strains MCC20^T (1472 bp), MCC57 (1418 bp), and their closely related type strains are shown, with *Kitasatospora setae* KM-6054^T (AB022868) serving as the outgroup. Bootstrap values greater than 50% from 1000 replications are displayed above the branches. The branch lengths are scaled to represent 0.0050 substitutions per nucleotide position.

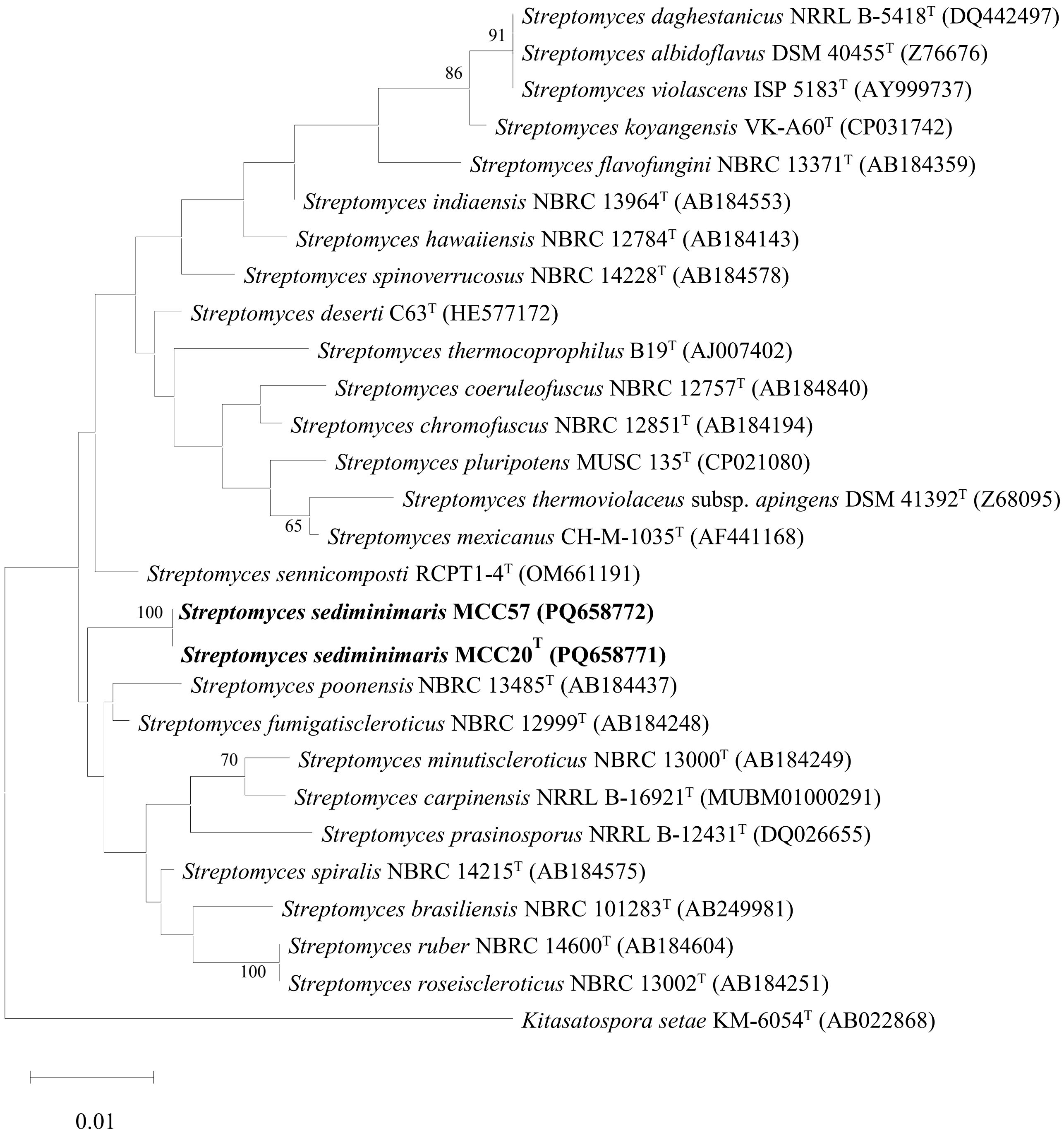

Fig. S2 16S rRNA gene-based phylogeny built by the Maximum-Likelihood (ML) algorithm. Genetic relationships among strains MCC20^T (1472 bp), MCC57 (1418 bp), and their closely related type strains are shown, with *Kitasatospora setae* KM-6054^T (AB022868) serving as the outgroup. Bootstrap values greater than 50% from 1000 replications are displayed above the branches. The branch lengths are scaled to represent 0.010 substitutions per nucleotide position.

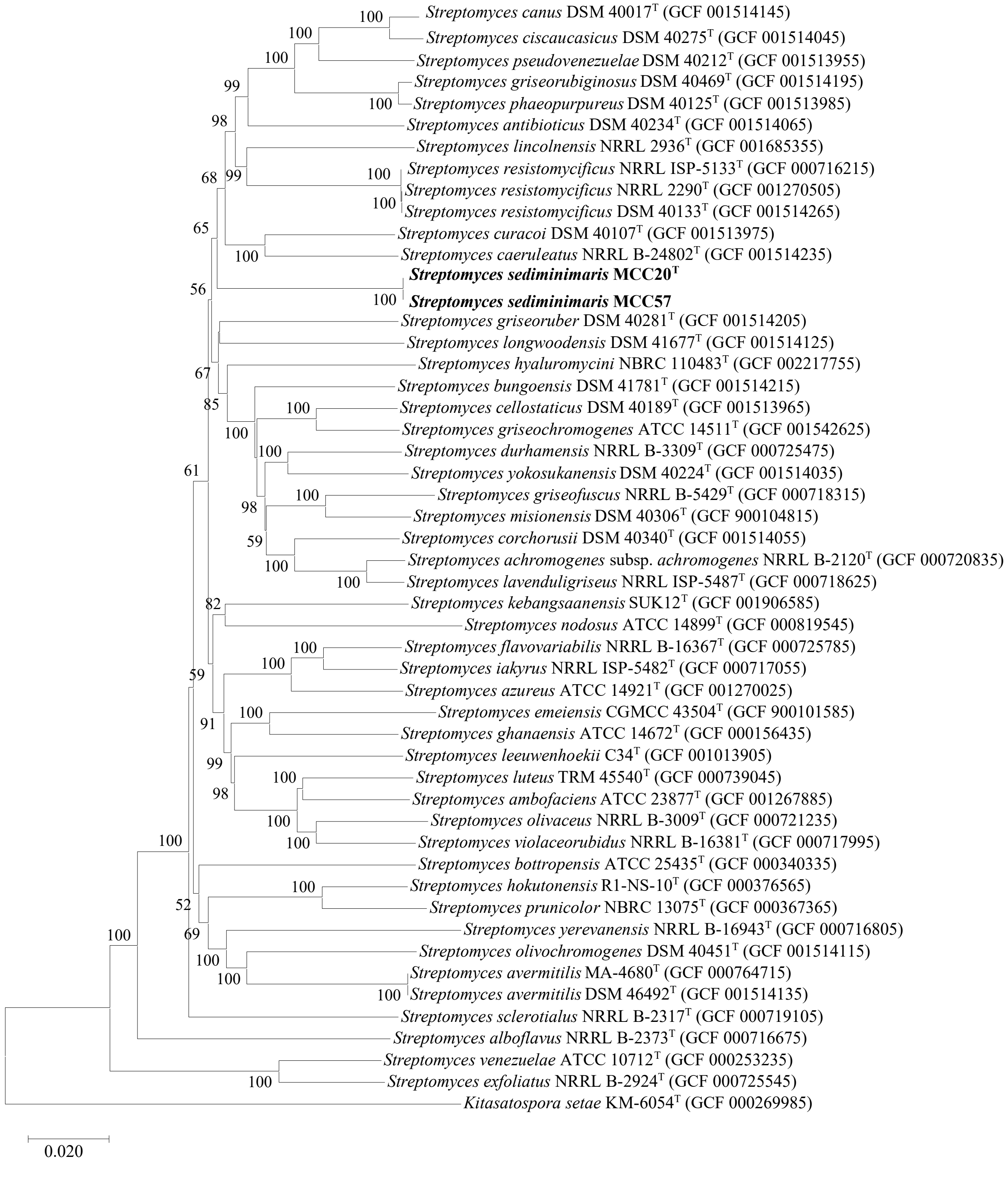

Fig. S3 Neighbor-joining tree based on multi-locus sequence analysis (MLSA) of 100 concatenated conserved gene sequences from strains MCC20^T, MCC57, and related taxa. *Kitasatospora setae* KM-6054^T (GCF 000269985) was used as the outgroup. Bootstrap values greater than 50% from 1000 replications, calculated using IQ-TREE ultrafast bootstrap analysis, are shown above the branches. Branch lengths are scaled to represent 0.020 nucleotide substitutions per site.

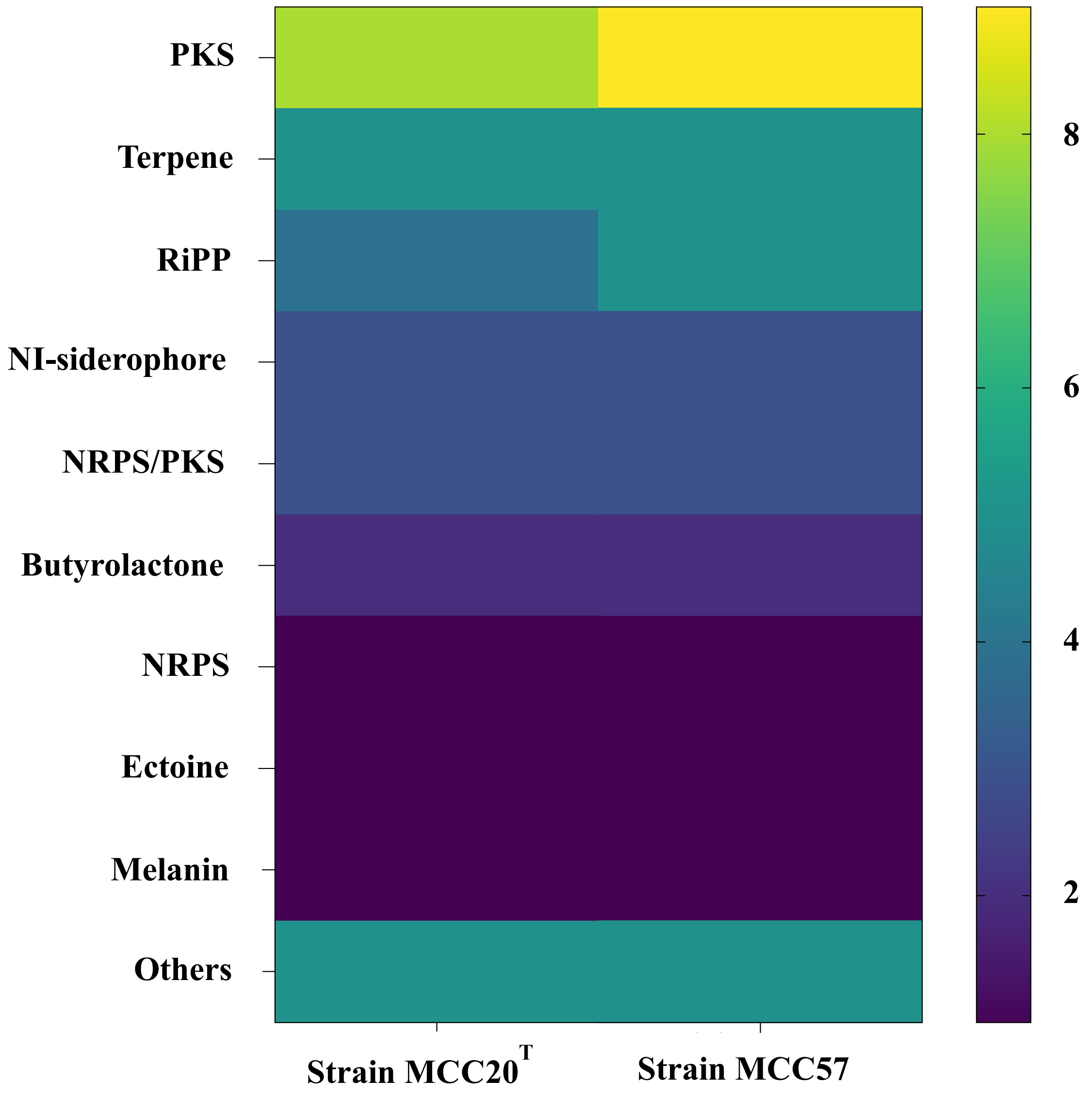

Fig. S4 Heatmap comparing the number of each biosynthetic gene cluster (BGC) type identified in the genomes of strains MCC20^T and MCC57.

Table S1. Colony appearances of strains MCC20^T, MCC57, and their closely related strains (14-day-old cultures).

Strain: 1, MCC20^T; 2, MCC57; 3, *S. fumigatiscleroticus* TBRC14820^T; 4, *S. spinosisporus* 7R016^T.

**Media**	**1**	**2**	**3**	**4***
**ISP2**				
Growth	Good	Good	Good	Good
Upper color	Light orange yellow-strong reddish orange	Strong reddish brown	Grayish yellow	Light yellow-green
Reverse color	Vivid orange	Pale orange yellow	Colorless	Pale greenish-yellow
Soluble pigment	Vivid yellow	Vivid yellow	Strong yellowish brown	None
Spore production	Poor (white)	None	None	Moderate (white)
**ISP3**				
Growth	Good	Good	Good	Good
Upper color	Light orange yellow-moderate reddish orange	Dark reddish brown	Dark orange yellow	Very pale green
Reverse color	Vivid orange yellow	Dark reddish brown	Yellowish white	Light yellow-green
Soluble pigment	Brilliant yellow	Vivid yellow	None	None
Spore production	Poor (white)	Poor (white)	None	Moderate (grey)
**ISP4**				
Growth	Good	Good	Good	Good
Upper color	Moderate-deep reddish orange	Strong-deep reddish orange	Light olive brown	Pale greenish-yellow
Reverse color	Deep yellowish pink	Strong reddish brown	Colorless	Strong greenish-yellow
Soluble pigment	Light yellow	Moderate yellow	None	None
Spore production	Poor (white)	None	None	Moderate (grey)

*Data are available in the supplementary materials of the online version of Kanchanasin P. et al., 2023 [1]

Table S1. Colony appearances of strains MCC20^T, MCC57, and their closely related strains (14-day-old cultures). (*continued*)

Strain: 1, MCC20^T; 2, MCC57; 3, *S. fumigatiscleroticus* TBRC14820 ^{T}; 4, *S. spinosisporus* 7R016^T.

Media	1	2	3	4*
**ISP5**				
Growth	Good	Good	Good	Good
Upper color	Moderate orange-strong reddish orange	Vivid orange-deep reddish orange	Moderate yellowish brown	Light yellow-green
Reverse color	Strong reddish orange	Pale yellow	Yellowish white	Pale orange-yellow
Soluble pigment	Pale yellow	Vivid yellow	Dark orange yellow	None
Spore production	None	None	none	Moderate (grey)
**ISP6**				
Growth	Good	Good	Good	Good
Upper color	Moderate orange yellow-dark reddish orange	Pale yellow-grayish reddish orange	Yellowish white	Light orange-yellow
Reverse color	Pale orange yellow	Pale yellow	Yellowish white	Brilliant orange-yellow
Soluble pigment	None	None	None	Yellow
Spore production	None	None	None	None
**ISP7**				
Growth	Good	Good	Good	Good
Upper color	Pale yellow-dark reddish orange	Pale orange yellow	Colorless	Light orange-yellow
Reverse color	Light-strong orange yellow	Light grayish yellowish brown	Colorless	Greyish-greenish-yellow
Soluble pigment	Dark orange yellow	None	Strong yellowish brown	None
Spore production	None	None	none	Moderate (grey)

*Data are available in the supplementary materials of the online version of Kanchanasin P. et al., 2023 [1]

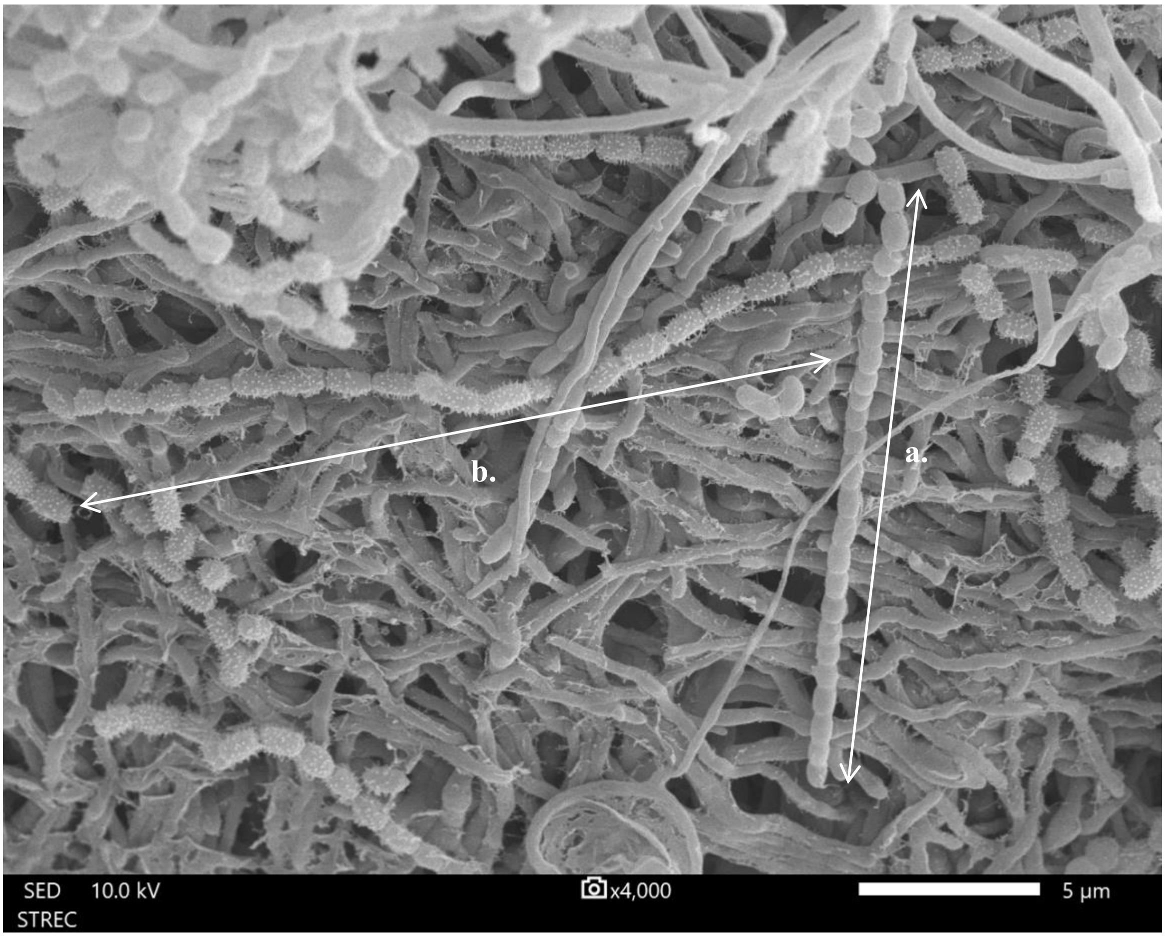

Fig. S5 Scanning electron micrograph visualizing the polysporous chains of strain MCC57 at (a.) early and (b.) mature stages of development after 14 days of incubation at 30°C on ISP3 medium. Bar, 5 µm.

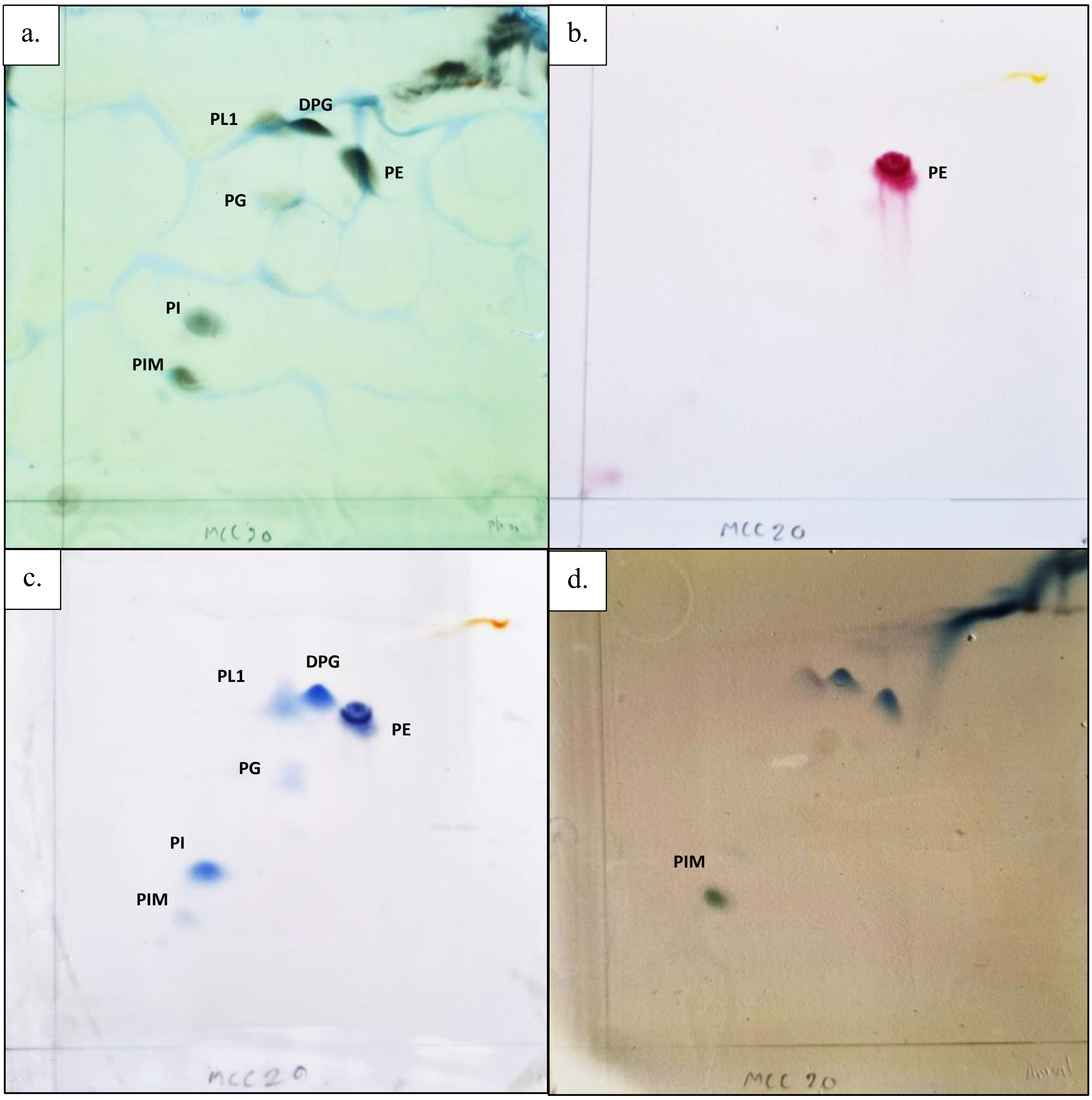

Fig. S6 Phospholipid pattern of strain MCC20^T analyzed using two-dimensional thin-layer chromatography (2D-TLC). The mobile phases were $CHCl_3$:CH_3OH:H_2O (65:25:4,v/v) for the 1st dimension and $CHCl_3$:CH_3COOH:CH_3OH:H_2O (40:7.5:6:2, v/v) for the 2nd dimension. Spots were visualized using the following reagents: a. 5.0% phosphomolybdic acid, b. 0.1% ninhydrin, c. Dittmer & Lester reagent, and d. anisaldehyde reagent. Phosphatidylcholine (PC) was not detected by Dragendorff's reagent.

Abbreviations: DPG, diphosphatidylglycerol; PE, phosphatidylethanolamine; PG, phosphatidylglycerol; PI, phosphatidylinositol; PIM, phosphatidylinositol mannoside; PL, unidentified phospholipid

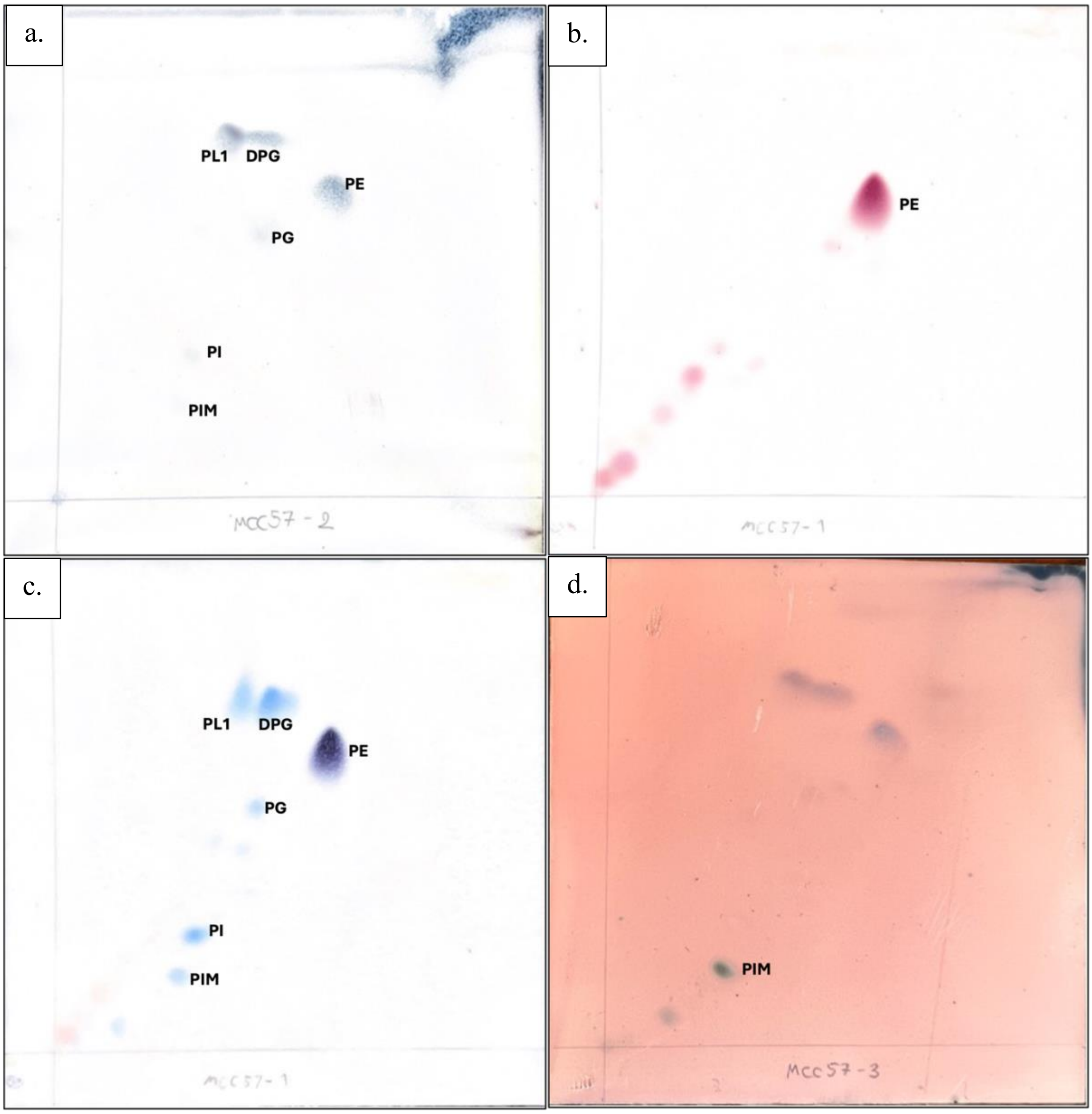

Fig. S7 Phospholipid pattern of strain MCC57 analyzed using two-dimensional thin-layer chromatography (2D-TLC). The mobile phases were $CHCl_3$:CH_3OH:H_2O (65:25:4, v/v) for the 1st dimension and $CHCl_3$:CH_3COOH:CH_3OH:H_2O (40:7.5:6:2, v/v) for the 2nd dimension. Spots were visualized using the following reagents: a. 5.0% phosphomolybdic acid, b. 0.1% ninhydrin, c. Dittmer & Lester reagent , and d. anisaldehyde reagent. Phosphatidylcholine (PC) was not detected by Dragendorff's reagent.

Abbreviations: DPG, diphosphatidylglycerol; PE, phosphatidylethanolamine; PG, phosphatidylglycerol; PI, phosphatidylinositol; PIM, phosphatidylinositol mannoside; PL, unidentified phospholipid

Table S2 Fatty acid profiles of strains MCC20^T and MCC57 compared with their closely related species.

Strain: 1, MCC20^T; 2, MCC57; 3, *S. fumigatiscleroticus* TBRC14820^T; 4, *S. spinosisporus* 7R016^T.

**Fatty acid***	**Fatty acid composition (%)**			
	**1**	**2**	**3**	**4**
$C_{16:0}$	6.5	3.7	2.5	5.4
$C_{20:0}$	–	3.3	1.5	–
iso-$C_{14:0}$	2.7	1.3	–	3.8
iso-$C_{15:0}$	16.8	9.7	14.1	16.3
anteiso-$C_{15:0}$	16.5	20.2	15.7	17.4
iso-$C_{16:0}$	22.4	18.5	18.9	24.0
iso-$C_{16:1}$H	3.3	3.3	2.2	2.3
iso-$C_{17:0}$	5.4	5.9	10.6	10.4
anteiso-$C_{17:0}$	8.4	16.0	18.8	12.5
anteiso-$C_{17:1}$ ω9c	2.9	5.2	3.9	1.8
iso-$C_{18:0}$	–	–	1.2	–
Summed Feature 3**	3.9	2.0	–	–
Summed Feature 9**	4.9	5.2	6.1	3.0

*Fatty acids contributing less than 1.0% are omitted.

**Summed Features represent groups of fatty acids that cannot be individually resolved under standard analytical conditions. The MIDI system reports these as a single feature with a combined percentage. Summed Feature 3 includes $C_{16:1}\omega$6c and/or $C_{16:1}\omega$7c, while Summed Feature 9 includes $C_{16:0}$ 10-methyl and/or *iso*-$C_{17:1}\omega$9c.

–, not detected

Supplementary reference

1. Kanchanasin P, Sripreechasak P, Suriyachadkun C, Rueangsawang K, Tanasupawat S, Phongsopitanun W. *Streptomyces cylindrosporus* sp. nov. and *Streptomyces spinosisporus* sp. nov.: two new endophytic actinobacteria isolated from the roots of *Barleria lupulina* Lindl. International Journal of Systematic and Evolutionary Microbiology 2023;73. DOI: https://doi.org/10.1099/ijsem.0.005926
